# Fluorinated small molecule derivatives in cancer immunotherapy: emerging frontiers and therapeutic potential

**DOI:** 10.3389/fimmu.2025.1622091

**Published:** 2025-07-18

**Authors:** Ka Fai Leong, Zihan Chen, Paolo Coghi

**Affiliations:** ^1^ Faculty of Chinese Medicine, Macau University of Science and Technology, Macau, Macau SAR, China; ^2^ School of Pharmacy, Macau University of Science and Technology, Macau, Macau SAR, China

**Keywords:** fluorinated small molecules, cancer immunotherapy, immune checkpoint inhibitors, STING agonists, fluorine in drug design, IDO1/TDO inhibitors

## Abstract

Immunotherapy has revolutionized cancer treatment by leveraging the body’s immune system to recognize and eliminate tumor cells. While monoclonal antibodies and checkpoint inhibitors have shown dramatic clinical successes, small molecules are increasingly recognized for their potential to modulate the immune system with improved pharmacokinetics and oral bioavailability. The incorporation of fluorine atoms into small molecule structures has become a widely used strategy to enhance therapeutic efficacy. Fluorine’s unique chemical properties such as high electronegativity, metabolic stability, and ability to modulate lipophilicity make fluorinated small molecules especially attractive for immunotherapeutic applications. This minireview highlights recent advances in fluorinated small molecules that target key immune pathways, including immune checkpoints, STING agonists, IDO inhibitors, and kinase pathways involved in immune regulation. We explore the chemical rationale, mechanisms of action, and therapeutic outcomes of fluorinated compounds currently in preclinical and clinical development. The discussion also addresses challenges such as immunotoxicity, resistance, and design strategies to overcome them. Together, these findings underscore the growing relevance of fluorinated small molecule immunotherapeutics in cancer treatment.

## 1 Introduction

The advent of immunotherapy has marked a paradigm shift in oncology, offering long-term remission for cancers that were once considered untreatable (1). Immune checkpoint inhibitors, such as those targeting PD-1, PD-L1, and CTLA-4, have led to breakthrough responses in several cancers. However, many patients fail to respond or develop resistance, underscoring the need for novel therapeutic modalities (2).

Small molecules are emerging as valuable alternatives or complements to antibody-based immunotherapies. Their advantages include oral administration, better tumor penetration, lower costs, and ability to target intracellular sites inaccessible to biologics (3–5). In this context, halogen plays a critical role in optimizing their performance.

Halogenation, particularly the replacement of hydrogen atoms with halogens, is a widely used strategy in compound optimization. Halogen substitution can sometimes enhance potency by orders of magnitude (6). Among halogens, fluorine substitution is especially notable for its profound impact on key physicochemical properties such as pKa, lipophilicity, metabolic stability, and molecular conformation. For instance, fluorination can alter the pKa exceed one log unit.

Fluorination usually increases lipophilicity, but may reduce it at saturated alkyl groups. Fluorine’s van der Waals radius, close to hydrogen’s, minimally affects conformation when monosubstitution. However, larger fluorinated groups such as trifluoromethyl, whose steric volume is comparable to that of an ethyl group, can significantly alter molecular geometry by changing bond angles (7, 8).

Fluorine substitution at chiral centers prone to *in vivo* racemization—such as that seen in thalidomide—has been shown to prevent such stereochemical interconversion. From a molecular interaction standpoint, fluorine forms weaker hydrogen bonds than hydrogen but exhibits stronger electrostatic interactions. Importantly, fluorine substitution at sites susceptible to metabolism by enzymes can confer metabolic resistance, primarily due to increased steric hindrance. This strategy is used in designing analog inhibitors, including nucleoside analogues (9–16).

This minireview focuses on the expanding role of fluorinated small molecule derivatives in cancer immunotherapy in last 5 years. We categorize recent developments into key target areas and highlight representative examples that illustrate the therapeutic promise of these compounds.

**Figure 1:**
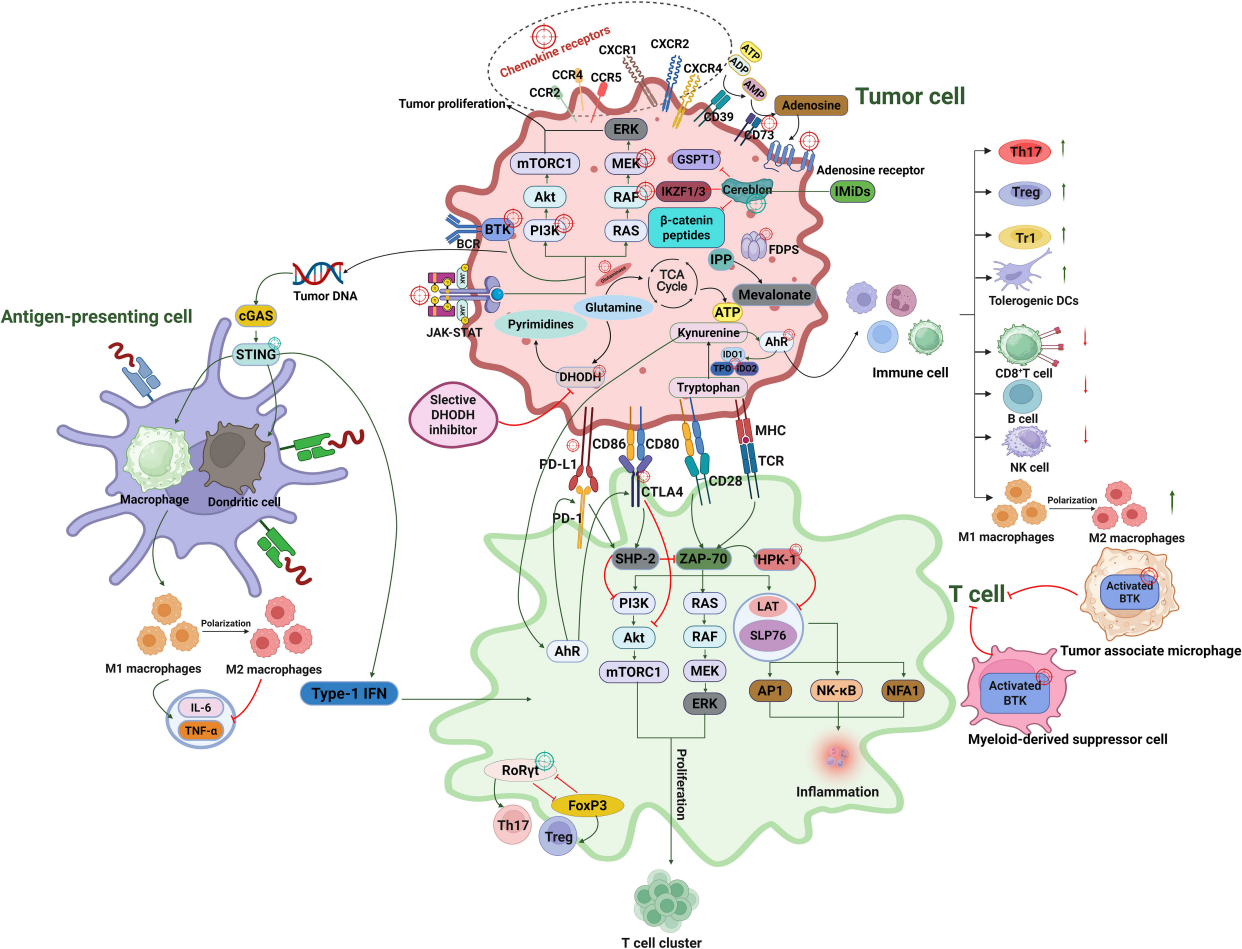
Integrated schematic of tumor immunomodulatory mechanisms involving metabolic, signaling, and checkpoint pathways across tumor cells, T cells, and antigen-presenting cells. The red crosshairs represent the suppression targets, and the green crosshairs represent the activation targets.

Here we present an integrated schematic (**Figure 1**) summarizing the key immunometabolic and signaling pathways that shape tumor–immune interactions and highlight therapeutic targets within the tumor microenvironment (TME).

This diagram illustrates key molecular pathways regulating the tumor-immune interface. In tumor cells, metabolic regulators such as DHODH, glutamine, pyrimidine synthesis, the TCA cycle, and tryptophan catabolism (via IDO/TDO) modulate immune escape and support proliferation. Transcriptional and signaling pathways including PI3K/AKT/mTOR, RAS/RAF/MEK/ERK, BTK, and β-catenin converge to sustain tumor cell survival, EMT, and resistance. Immunomodulatory protein degradation by cereblon–recruited E3 ligases targets substrates such as IKZF1/3, GSPT1, and β-catenin fragments, influencing immune regulation and tumor viability. Tumor-derived chemokines (CCL2, CCL5, CCL22, CXCL8, CXCL12) recruit suppressive immune cells including tumor-associated macrophages (TAMs), myeloid-derived suppressor cells (MDSCs), and regulatory T cells (Treg) via chemokine receptors (CCR2, CCR4, CCR5, CXCR1/2, CXCR4), reinforcing an immunosuppressive TME. The STING–cGAS pathway in antigen-presenting cells, macrophages, dendritic cells (DCs), activates type I interferon responses that prime cytotoxic T cell activity. T cell activation is mediated through TCR/CD28 costimulation, engaging PI3K, LAT, and MAPK cascades; this is negatively regulated by immune checkpoints (PD-1/PD-L1, CTLA-4/CD80) and intracellular suppressors such as HPK1. T cell lineage commitment is modulated by transcriptional regulators including FoxP3 and RORγt, influencing Treg and Th17 differentiation, respectively. This figure synthesizes multiple molecular interactions to illustrate how tumor cells co-opt immune regulatory pathways and highlights potential therapeutic targets across immune metabolism, checkpoint inhibition, signal transduction, and transcriptional control.

## 2 Class of compounds

**Figure 2:**
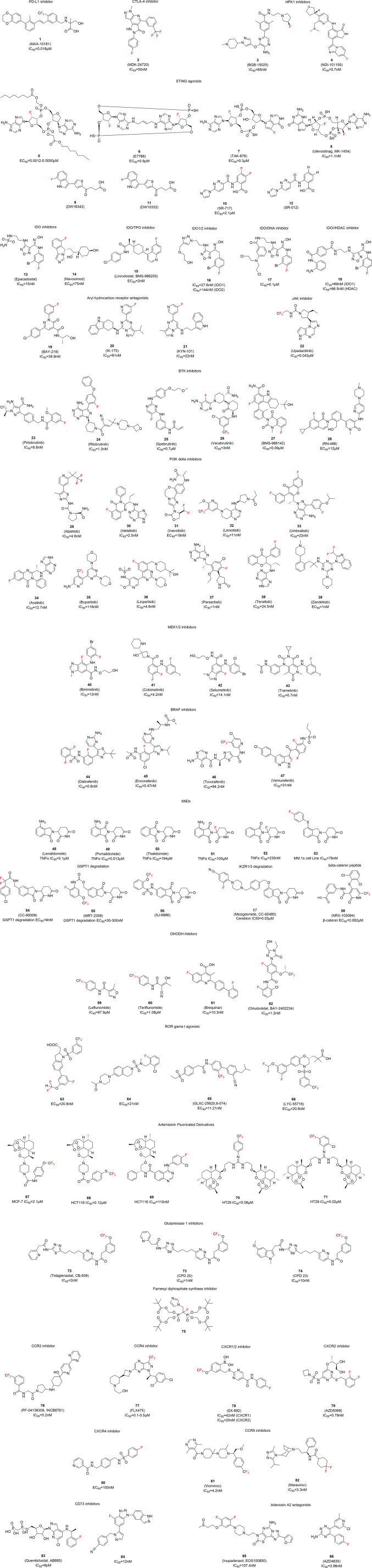
Fluorinated compounds with immunomodulatory activity.

We identified over 80 fluorinated small molecules with reported immunomodulatory activity in the past five years. Their chemical structures are illustrated in **Figure 2** and are categorized according to their pharmacological mechanisms.

### 2.1 Fluorinated immune checkpoint inhibitors

Immune checkpoints serve as critical regulators that suppress T cell activity to maintain immune homeostasis. However, tumors hijack these pathways to evade immune surveillance. Checkpoint blockade therapies, such as anti-PD-1 and anti-CTLA-4 antibodies, have revolutionized cancer treatment by reactivating antitumor immunity. Nonetheless, challenges such as immune-related toxicities, poor tumor penetration, and therapeutic resistance necessitate the exploration of alternative strategies.

Small molecule immune checkpoint inhibitors (ICIs) offer potential advantages, including oral bioavailability, improved tissue penetration, and cost-effective production (17, 18). Their development is crucial for broadening the scope of immunotherapy across different cancer types and patient populations.

#### 2.1.1 Small molecule PD-1/PD-L1 inhibitors

While most approved PD-1/PD-L1 inhibitors are monoclonal antibodies, small molecule alternatives are under development to overcome limitations such as poor tissue penetration and immune-related adverse events (irAEs) (19, 20).

Fluorinated aromatic groups have been critical in enhancing the binding affinity of small molecule PD-L1 inhibitors. Compounds such as compound 1 (21, 22), and others utilize fluoroaryl moieties to improve hydrophobic interactions within the PD-L1 binding pocket. For instance, a fluorophenyl group in the BMS series increases π-stacking and binding site complementarity, enhancing antagonistic activity.

#### 2.1.2 Small molecule CTLA-4/CD80 inhibitors

While less explored than PD-1/PD-L1, fluorinated derivatives compound 2 targeting the CTLA-4/CD80 axis are being investigated (23). These molecules often feature fluorinated indole or quinazoline cores to mimic protein-protein interaction surfaces.

#### 2.1.3 Small molecule HKP1 inhibitors

Hematopoietic progenitor kinase 1 (HPK1) is an immunosuppressive regulatory factor that is initially expressed at low levels in lymphoid progenitor cells but becomes highly expressed in mature immune cells, such as T cells, B cells, DCs, and M2 macrophages (24, 25). Inhibiting HPK1 has emerged as a promising strategy to enhance antitumor immunity.

Several fluorinated small molecule HPK1 inhibitors have already progressed into clinical trials, including compound 3 and 4 (26–29). These inhibitors aim to counteract HPK1-mediated suppression of T cell receptor (TCR) signaling, thus potentiating T cell activation and improving the efficacy of cancer immunotherapies.

Fluorine in HPK1 inhibitors improves binding, stability, and pharmacokinetics, enabling synergy with checkpoint inhibitors for cancer therapy.

### 2.2 STING agonists with fluorinated enhancements

The STING (Stimulator of Interferon Genes) pathway activates innate immunity via type I interferon responses, making it a promising target for cancer immunotherapy. While many STING agonists are locally administered due to systemic toxicity, fluorinated analogs have shown potential for oral or systemic use by improving pharmacokinetic profiles.

#### 2.2.1 Cyclic dinucleotide mimetics

Natural STING agonists, such as cyclic dinucleotides (CDNs), suffer from poor membrane permeability and metabolic degradation. Fluorinated analogs have been synthesized to address these limitations (30).

For example, fluorinated deoxyribose and indole-based STING agonists have demonstrated improved potency, cellular uptake, and resistance to enzymatic hydrolysis. Strategic fluorine placement stabilizes the molecule and enhances cGAS-STING pathway activation in tumor-associated immune cells (31).

Dejmek and colleagues substituted fluorine atoms for the free hydroxyl groups on the pentose ring of cyclic dinucleotides. Remarkably, this modification increased the agonistic activity by 10- to 100-fold. The enhanced efficacy is attributed to better cell permeability, greater anti-degradation properties, and stronger binding to the STING protein.

To further improve cellular permeability and metabolic stability, long-chain alkyl groups were conjugated to the phosphate hydroxyl groups to create prodrugs. For instance, compound 5, modified with an n-octanoyl group, exhibited an EC_50_ of less than 1 nM, representing more than a 10,000-fold improvement in potency compared to the parent CDN (32).

Several fluorinated CDN analogs, such as compound 6-8 (33–39), have advanced into phase I clinical trials, further underscoring the translational potential of this approach.

#### 2.2.2 Non-nucleoside STING activation

Non-nucleoside drugs are also a research hotspots. Compared with traditional CDN STING agonists, non-nucleoside drugs have better metabolic stability and cell permeability. Some compounds are orally bioavailable. However, their development is hindered by structural complexity and species-specific STING binding differences between humans and models like mice.

Compound 9 (40) and 10 (41) are particularly noteworthy. Compared with its non-fluorinated analog compound 11, compound 9 incorporates fluorine at the 7-position of the indole ring, significantly improving binding stability, metabolic resistance, and, to a lesser extent, agonistic activity. Similarly, compound 10 demonstrates the crucial role of fluorine in modulating bioactivity: its non-fluorinated precursor, compound 12, exhibits no STING activation, whereas the fluorine-substituted compound 10 shows strong thermal stability and induces a closed conformation of the STING protein, effectively triggering downstream immune responses.

### 2.3 IDO and TDO pathway inhibitors

Indoleamine 2,3-dioxygenase (IDO1) and tryptophan 2,3-dioxygenase (TDO) enzymes mediate immune suppression in the TME by depleting tryptophan and producing immunosuppressive kynurenines (42–44). These pathways impair T cell proliferation, promote Treg differentiation, and support tumor immune escape.

#### 2.3.1 Fluorinated IDO1 inhibitors

Fluorinated derivatives of IDO1 inhibitors have demonstrated improved pharmacological properties, including enhanced metabolic stability, target specificity, and bioavailability. Compounds such as compound 13 and 14 (45–47) incorporate fluoroaryl groups to fine-tune physicochemical properties such as pKa and lipophilicity, optimizing enzyme inhibition under physiological conditions. These modifications improve selectivity and reduce off-target toxicity, critical for clinical success.

#### 2.3.2 Dual inhibition

To overcome redundancy between the IDO and TDO pathways, dual inhibitors are under development. Many of these compounds incorporate fluorinated aromatic rings that span both enzyme binding pockets, enhancing dual target engagement. One notable example is compound 15 (47, 48), a fluorinated dual IDO/TDO inhibitor with promising clinical activity.

Beyond IDO/TDO dual inhibitors, other bifunctional molecules have been designed to target IDO1 along with additional oncogenic or epigenetic pathways. For example, compound 16, a derivative of epacadostat 13, inhibits both IDO1 and IDO2 (49), compound 17 possesses both IDO1 inhibitory and DNA alkylation activities (50), and compound 18 simultaneously inhibits IDO1 and histone deacetylases (HDACs), which modulate chromatin architecture and gene expression (51). These inhibitors may enhance antitumor efficacy via synergy.

#### 2.3.3 Aryl hydrocarbon receptor antagonists

Kynurenine binds to the aryl hydrocarbon receptor (AhR) and further suppresses antitumor immunity (52, 53). Fluorinated AhR antagonists—such as compound 19-21 (54–57)—can block this interaction, thereby restoring immune activation and reprogramming the TME. Fluorine incorporation into these molecules enhances their metabolic stability and binding affinity, which are essential for disrupting the kynurenine-AhR axis and achieving therapeutic benefit.

### 2.4 Kinase inhibitors with immunomodulatory functions

Many oncogenic kinases also regulate immune evasion and cytokine production, making kinase inhibitors effective immunomodulatory agents.

#### 2.4.1 JAK/STAT pathway

Fluorinated Janus kinase (JAK) inhibitors, such as compound 22 (approved in 2019), modulate interferon signaling and T-cell responses. Fluorination enhances selectivity among JAK isoforms and reduces hematologic toxicity (58).

#### 2.4.2 BTK and PI3K inhibitors

Bruton’s tyrosine kinase (BTK) and phosphoinositide 3-kinase delta (PI3K-δ) inhibitors influence B-cell malignancies and Treg populations. Fluorinated analogs exhibit improved target occupancy and immune reprogramming effects.

As of April 2025, five BTK inhibitors have been FDA-approved (59–63), two of which—compound 23,24—are fluorinated (64, 65). Other fluorinated BTK inhibitors, such as compound 25-28 (66–74), are under clinical or preclinical investigation.

Regarding PI3K-δ inhibitors, seven have received FDA approval (75–81), five of which—compound 29-33—are fluorinated (82–86). Additional fluorinated PI3K-δ inhibitors under clinical development include compound 34-39 (86–96) with most having completed phase II trials.

#### 2.4.3 BRAF and MEK1/2 inhibitors

The RAF/MEK/ERK pathway regulates tumor cell proliferation, differentiation, survival, and migration, and it also modulates immune responses. ERK activation increases immunosuppressive factors, such as interleukin-10 (IL-10), promotes Th2 cell differentiation, and reduces CD8^+^ T-cell and B-cell activity (97).

Currently, five MEK1/2 inhibitors are FDA-approved (98–106), four of which——compound 40-43——are fluorinated. Additionally, all approved BRAF inhibitors, compound 44-47, are fluorinated agents (107–111).

### 2.5 Fluorinated immunomodulators on hematopoietic system and immune cell differentiation

#### 2.5.1 Fluorinated derivatives of thalidomide and lenalidomide

Glutarimide-based immunomodulatory drugs (IMiDs), such as lenalidomide 48, pomalidomide 49 and thalidomide 50, modulate cereblon, a component of the E3 ubiquitin ligase complex, to induce selective protein degradation (112, 113). IMiDs enhance T cell and NK cell activities by promoting IL-2, CD28, and IFN-γ expression. Their combination with monoclonal antibodies like daratumumab or elotuzumab is recommended as first-line therapy for multiple myeloma (114). IMiDs are also under investigation in combination with CAR-T therapy and cancer vaccines such as PVX-410 (115). Fluorination of the phthalimide or glutarimide ring, compound 51-53, can fine-tune cereblon binding and degradation selectivity, thus providing a stronger immunomodulation effect (116, 117).

New-generation IMiDs have improved specificity to minimize side effects. Examples include compound 54,55 targeting GSPT1, compound 56 targeting GSPT1/2, compound 57 targeting IKZF1/3, and compound 58 targeting β-catenin peptides (118–124). G_1_ to S phase transition 1/2 (GSPT1/2) are a key factor in cell proliferation and Ikaros zing-finger family transcription factors (IKZF TFs) are a regulators play an important role in lymphocyte development and differentiation (125). In the other hand, β-catenin peptide is a key transducer in Wnt signal pathway, has a very close relationship with tumor occurrence (126).

#### 2.5.2 DHODH inhibitor

Dihydroorotate dehydrogenase (DHODH) is a mitochondrial enzyme responsible for the fourth step of pyrimidine biosynthesis and is essential for cellular proliferation. Currently available fluorinated DHODH inhibitors include leflunomide 59 and its active metabolite teriflunomide 60, primarily used to treat rheumatoid arthritis and multiple sclerosis (127–129). Although these drugs inhibit T and B cell proliferation, many cancer cells upregulate DHODH expression to sustain rapid growth. Compounds such as compound 61,62 (129–132) have entered early-phase clinical trials targeting acute myeloid leukemia.

#### 2.5.3 Immune cell differentiation

Retinoic acid receptor-related orphan receptor gamma t (RORγt) is a nuclear transcription factor specific to immune cells. Its activation induces the differentiation of T cells into Th17 cells, thereby enhancing antitumor immunity (133). Several fluorinated small molecules targeting RORγt have been reported, including Compound 63-66 (134–138), the latter having advanced into clinical trials.

### 2.6 Fluorinated molecules in tumor microenvironment modulation

The tumor microenvironment (TME) plays a central role in immune suppression. Fluorinated small molecules can modulate several aspects of the TME:

#### 2.6.1 pH sensitive and iron sensitive fluorinated compound

pH-responsive fluorinated carriers enhance drug release in acidic tumor conditions. Fluorination improves nanoparticle stability and pH sensitivity without impairing self-assembly (139). These nanoparticles preferentially release drugs in acidic TME (pH 5.0–6.5), reducing systemic toxicity at physiological pH (~7.4).

Additionally, acidic conditions promote iron release, and tumor cells upregulate transferrin receptor 1 (TfR1) to meet proliferative demands (140). Artemisinin, leveraging its endoperoxide bridge, generates reactive oxygen species (ROS) in high-iron environments, inducing tumor cell death. Fluorinated derivatives of artemisinin, compounds 67-71, show improved cell penetration, ROS generation, stability, and tumor selectivity (141–144).

#### 2.6.2 Metabolic inhibitors

Fluorinated metabolic inhibitors block lactate production or glutaminolysis, reprogramming immune cell metabolism. Metabolic reprogramming in tumors, such as increased lactate production and glutaminolysis, contributes to an immunosuppressive microenvironment. Targeting these pathways can modulate immune responses (145).

While specific fluorinated inhibitors are under investigation, the general strategy involves disrupting lactate dehydrogenase activity and glutamine metabolism to reprogram immune cell function within tumors (146). Notable drug candidates under clinical investigation include compound 72 and its preclinical analogues compound 73,74 (147–149).

In addition to targeting lactate and glutamine, inhibition of farnesyl diphosphate synthase (FDPS) is another emerging area of research. FDPS is a key enzyme in the mevalonate pathway, responsible for depleting isopentenyl pyrophosphate (IPP), thereby reducing T cell activation and impairing antigen presentation by DCs and macrophages. FDPS also activates osteoclasts, promoting tumor metastasis. Bisphosphonates like zoledronate target FDPS to reduce bone destruction. Fluorination of bisphosphonates, compound 75, significantly enhances their inhibitory potency, increases T cell activity—particularly Vγ2Vδ2 T cells—improves antigen presentation, and reduces bone metastasis (150).

#### 2.6.3 Chemokine receptor

Chemokine receptors play critical roles in tumor growth, metastasis, and immune suppression (151), Several fluorinated chemokine receptor antagonists have been developed, targeting CCR2 (152), CCR4 (153), CCR5 (154), CXCR1 (155), CXCR2 (156), and CXCR4 (157, 158), effectively blocking immune cell trafficking that promotes tumor growth.

Multiple chemokine receptor antagonists have advanced to clinical trials, including compound 76 targeting CCR2 (159, 160), compound 77 targeting CCR4 (161–164), compound 78, a dual CXCR1/2 inhibitor (165, 166), compound 79 targeting CXCR2 (156, 167), Compound 80 targeting CXCR4 (157), and the repurposed HIV drugs vicriviroc 81 and maraviroc 82 (168–170) as CCR5 antagonists.

#### 2.6.4 Fluorinated CD39/CD73/A2AR antagonists

Fluorinated CD39/CD73/A2AR antagonists can block tumors from exploiting high levels of ATP in the TME to establish an immune-cold shield and promote tumor growth. CD39 converts extracellular ATP and ADP into AMP, while CD73 further converts AMP into adenosine, which activates the adenosine 2A receptor (A2AR), promoting the expansion of immunosuppressive cell types such as Treg and Th2 cells (171).

Reported fluorinated CD73 inhibitors include compound 83 (172, 173) and a compound 84 discovered by Beatty et al. (174). Among A2AR antagonists, compound 85,86 (175–178) have entered clinical trials.

## 3 Discussion and future prospective

Fluorine incorporation in small molecule immunotherapeutics enhances potency, selectivity, and durability. In checkpoint inhibition, it improves PD-L1 and CTLA-4 antagonist properties. Fluorinated STING agonists show better uptake and activity, while IDO1 and TDO inhibitors gain improved pharmacokinetics and immune modulation, advancing cancer immunotherapy strategies.

Though promising for immunotherapy due to enhanced pharmacokinetic properties and specificity, fluorinated small molecules also present challenges, notably the risk of irAEs stemming from systemic immune activation (179). Although direct studies linking fluorinated compounds to irAEs are limited, insights can be drawn from the broader context of immunotherapies, particularly ICIs, which have been extensively studied for their irAE profiles (180, 181).

Researches indicate that ICIs disrupt immune balance, causing irAEs via multiple mechanisms. For instance, a comprehensive review highlights that the activation or reactivation of T cells is a dominant factor in the development of ICI-related irAEs, with enhanced Th17 cell responses contributing to the production of proinflammatory cytokines like IL-17A, IL-21, and IL-22 (179). These findings underscore the delicate balance required in immunotherapy: enhancing anti-tumor immunity while minimizing collateral immune activation that can lead to adverse events.

Fluorinated small molecules for immunotherapy may trigger irAEs. Mitigation strategies include dose optimization, immune pathway modulation, and thorough preclinical testing. Off-target effects and metabolite toxicity also warrant attention. Future designs should enhance selectivity, reduce toxicity, and address resistance mechanisms like immune suppression or compensatory signaling.

To overcome these challenges, future design strategies include:

Structure-guided drug design incorporating fluorine in metabolically vulnerable regions to enhance stability.

Proteolysis targeting chimera and molecular glues with fluorinated ligands to achieve targeted protein degradation (119).

Nanoformulations and prodrugs using fluorinated linkers to control release and reduce systemic exposure.

Companion diagnostics, such as fluorine-18 labeled PET tracers, to visualize immune response and guide therapy.

The integration of AI-driven molecule optimization, high-throughput screening, and deep learning models for ADMET prediction will further accelerate the development of next-generation fluorinated immunotherapeutics (182).

In summary, fluorinated small molecules represent a dynamic and growing class of agents in cancer immunotherapy. By merging the principles of fluorine chemistry with immuno-oncology, researchers can design novel agents capable of overcoming existing limitations and ushering in a new era of precision immunotherapy.
